# Case Report: A Rare Case of Autoinflammatory Phospholipase Cγ2 (PLCγ2)-Associated Antibody Deficiency and Immune Dysregulation Complicated With Gangrenous Pyoderma and Literature Review

**DOI:** 10.3389/fimmu.2021.667430

**Published:** 2021-05-19

**Authors:** Na Wu, Bingqing Zhang, Tao Wang, Min Shen, Xuejun Zeng

**Affiliations:** ^1^Department of Rheumatology, State Key Laboratory of Complex Severe and Rare Diseases, Peking Union Medical College Hospital, Chinese Academy of Medical Sciences & Peking Union Medical College, National Clinical Research Center for Dermatologic and Immunologic Diseases (NCRC-DID), Key Laboratory of Rheumatology and Clinical Immunology, Ministry of Education, Beijing, China; ^2^Department of General Internal Medicine, Peking Union Medical College Hospital, Chinese Academy of Medical Science & Peking Union Medical College, Beijing, China; ^3^Department of Dermatology, Peking Union Medical College Hospital, Chinese Academy of Medical Sciences & Peking Union Medical College, Beijing, China

**Keywords:** autoinflammatory disease, autoinflammatory phospholipase Cγ2-associated antibody deficiency and immune dysregulation syndrome, gangrenous pyoderma, phospholipase Cγ2, hyperimmunoglobulinemia E, TNFα inhibitor

## Abstract

**Background:**

Autoinflammatory phospholipase Cγ2 (PLCγ2)-associated antibody deficiency and immune dysregulation (APLAID) is a rare autoinflammatory disease caused by gain-of-function mutations in the *PLCG2* gene. Here we report a rare case of APLAID patient carrying a novel heterozygous missense *PLCG2* I169V mutation with gangrenous pyoderma and concomitant high serum immunoglobulin (Ig) E level.

**Methods:**

The patient was diagnosed as APLAID and has been treated in our department. His phenotype and genotype were carefully documented and studied. We also conducted a comprehensive literature review on APLAID.

**Results:**

A 23-year-old Chinese Han man presented with recurrent fever for 18 years and vesiculopustular rashes for 9 years, along with chronic bronchitis, leukocytosis, increased C-reactive protein, immunodeficiency and high serum IgE. Skin biopsy showed chronic inflammatory cells infiltration. A paternal heterozygous missense variant in exon 6 of the *PLCG2* gene p. I169V was identified. His vesiculopustular and IgE level responded to medium dose corticosteroids. After withdrawal of steroids, he developed severe arthritis and a large deteriorating ulceration resembling pyoderma gangrenosum on the left knee. Large dose corticosteroids were suboptimal. Then he received adalimumab with satisfactory response for arthritis and skin lesion. But he got an immunodeficiency-associated lymphoproliferative disorder 2 months later. Through literature review, there were a total of 10 APLAID patients reported by six English-language publications. Vesiculopustular rashes, sinopulmonary infection and immunodeficiency were the most frequent symptoms of APLAID patients. Glucocorticoids, intravenous immunoglobulin and biologics were clinically used to treat APLAID but none of these patients had a complete recovery.

**Conclusions:**

The rarity and diversity of APLAID make it difficult to be diagnosed. Our study reported the first case of APLAID with gangrenous pyoderma and concomitant high IgE carrying a novel *PLCG2* mutation, which may expand the clinical phenotype and genotype of APLAID.

## Introduction

Autoinflammatory diseases are a group of inherited conditions due to defects in genes that regulate innate immunity, characterized by systemic inflammation and the lack of high-titer autoantibodies or antigen-specific T lymphocytes (1, 2). The spectrum of systemic autoinflammatory disorders broadens continually owing to the advances in genetic sequencing techniques (3). Autoinflammatory phospholipase Cγ2 (PLCγ2)-associated antibody deficiency and immune dysregulation (APLAID, OMIM 614878) is a rare autoinflammatory disease caused by gain-of-function mutations in the *PLCG2* gene (OMIM 600220). *PLCG2* is located in chromosome 16, and encodes PLCγ2, an enzyme with a regulatory function in immune and inflammatory pathways, which is highly expressed in hematopoietic cells, including B cells, natural killer (NK) cells, mast cells and macrophages. PLCγ2 can hydrolyze the substrate phosphatidylinositol 4,5-bis-phosphate (PIP_2_) to generate diacylglycerol (DAG) and inositol 1,4,5-trisphosphate (IP_3_). IP_3_ functions as a second messenger to increase intracellular calcium concentration, inducing downstream cell activities (4–6). In 2012, Zhou et al. first reported a father and his daughter presented with early-onset recurrent skin inflammation and granulomata, nonspecific interstitial pneumonitis with respiratory bronchiolitis (NSIP), arthralgia, eye inflammation, enterocolitis, cellulitis, and mild immunodeficiency (7). A substitution in the *PLCG2* gene (NM_002661.3) c.2120C>A, p.Ser707Tyr was confirmed, and this mutation located at the SH2 domain was found to create a novel signaling receptor site, resulting in hyper-reactive protein and constitutively activated down-stream signaling pathways (7).

Until now, only a few cases of APLAID have been reported in English literature. Here, we report the first case of a Chinese patient with APLAID caused by a novel *PLCG2* gene mutation with rare manifestation of gangrenous pyoderma and concomitant high serum immunoglobulin (Ig) E level, which has not been reported before. We also reviewed the published English literature of APLAID.

## Methods

The patient was referred to and followed up in our tertiary medical center. Complete medical records and detailed data were collected and documented. Whole exome sequencing by Next Generation Sequencing was performed in the Center for Genetic Testing, MyGenotics Medical Laboratory, Beijing, China. We performed a systematic literature search in PubMed using the terms as “APLAID” OR “autoinflammatory phospholipase Cγ2 (PLCγ2)-associated antibody deficiency and immune dysregulation”. After screening, six articles containing a total of 10 cases of APLAID patients were reviewed.

This research was approved by the Institutional Review Board of Peking Union Medical College Hospital and performed according to the Declaration of Helsinki. Informed consent was obtained from the participant.

## Results

### Case Presentation

**Figure 1:**
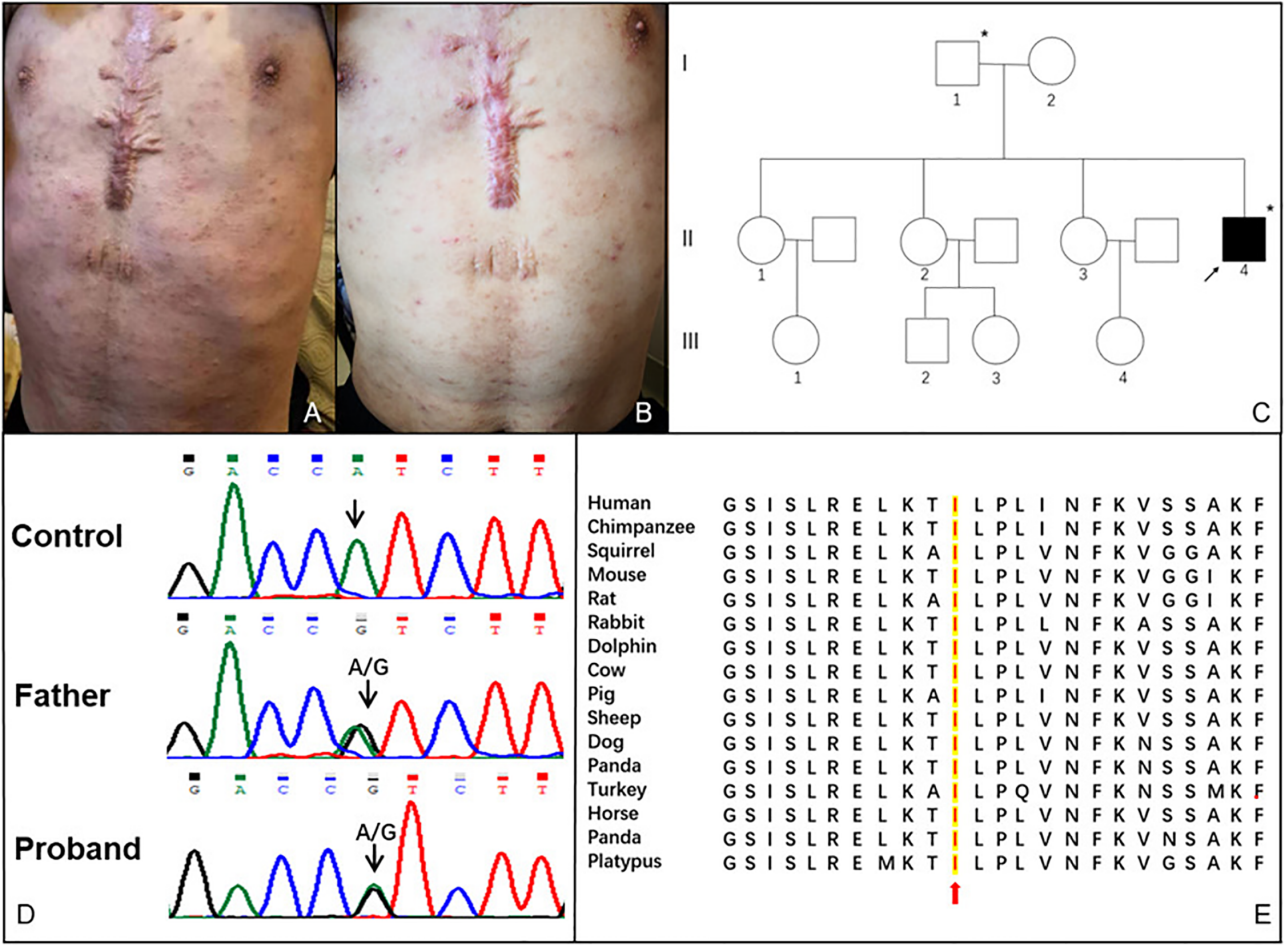
**(A)** Vesiculo-pustular rashes on the trunk of this patient before treatment; **(B)** Rashes on the trunk of this patient after treatment; **(C)** Pedigree of the patient. The arrow indicates the proband. The asterisks indicate the individuals who carried c.505A > G (p. I169V) mutation in the exon 6 of the *PLCG2* gene and presented with high immunoglobulin E; **(D)** Sanger sequencing result of the *PLCG2* gene from the proband and his father; **(E)** Sequence alignment of PLCγ2 protein among various vertebrates.

A 23-year-old Chinese Han man was presented with recurrent fever for 18 years and vesiculopustular rashes for 9 years. He started to have recurrent low-grade fever since 5 years old. Each episode lasted 2 to 3 days and repeated every several months. No accompanying symptoms were noticed then and it stopped at the age of 7 years. At the age of 14 years, painless and non-itching vesiculopustular rashes were noticed all-over his body (**Figure 1A**), which lasted about 2 to 3 weeks. The rashes deteriorated with high fever since 19 years old. Such episodes relapsed every 3 to 4 months. At initial presentation, he was febrile with widespread vasiculopustular rashes, accompanied with headache, cough and lower limb swelling. There were no oral ulcers, conjunctivitis, chest pain, abdominal pain, diarrhea, myalgia or hearing loss. No food or drug allergy was reported. The patient underwent surgical repair of atrial septal defect at the age of 7. He denied family history of autoinflammatory diseases (**Figure 1C**).

White blood cell counts increased during the flares and normalized during the intervals. Eosinophils, hemoglobin, and platelets counts were normal. C-reactive protein elevated persistently, while erythrocyte sedimentation rate was in the normal range. The biochemistry panel and routine urine analysis were normal. Laboratory data showed decreased serum levels of IgA [0.09 g/L; reference range (RR):0.7–4.0], IgG (6.08 g/L; RR: 7–16), and IgM (0.07 g/L; RR: 0.4–2.3), while significantly increased level of IgE (1781–2082 KU/L; RR: 0–60). B cell counts decreased to 51/µL (RR: 90–660), and NK cell counts decreased to 15/μL (RR: 46–590). Testing for antinuclear antibodies (ANAs) and anti-neutrophil cytoplasmic antibodies (ANCA) were negative. EBV-DNA and CMV-DNA were within normal levels. Ultrasound imaging revealed superficial lymph nodes enlargement and splenomegaly. Computerized tomography demonstrated chronic inflammatory bronchial wall thickening of the bilateral lower lobes and lympho-splenomegaly. A skin biopsy showed superficial dermis infiltration by chronic inflammatory cells. Whole exome sequencing identified a novel paternal heterozygous missense mutation in exon 6 of the *PLCG2* gene c.505A>G, p.Ile169Val, which is a highly conserved residue (**Figures 1D, E****)**. However, his father was symptom-free except for high serum IgE level (163 KU/L). He was treated with prednisone 30 mg per day, methotrexate 12.5 mg per week and intravenous immunoglobulin (IVIG) 20 g per day once for 3 days every 3 months, with a satisfactory response for the fever, rashes (**Figure 1B**), bronchitis and headache. Steroid was tapered consequently and methotrexate was stopped for intolerance.

**Figure 2:**
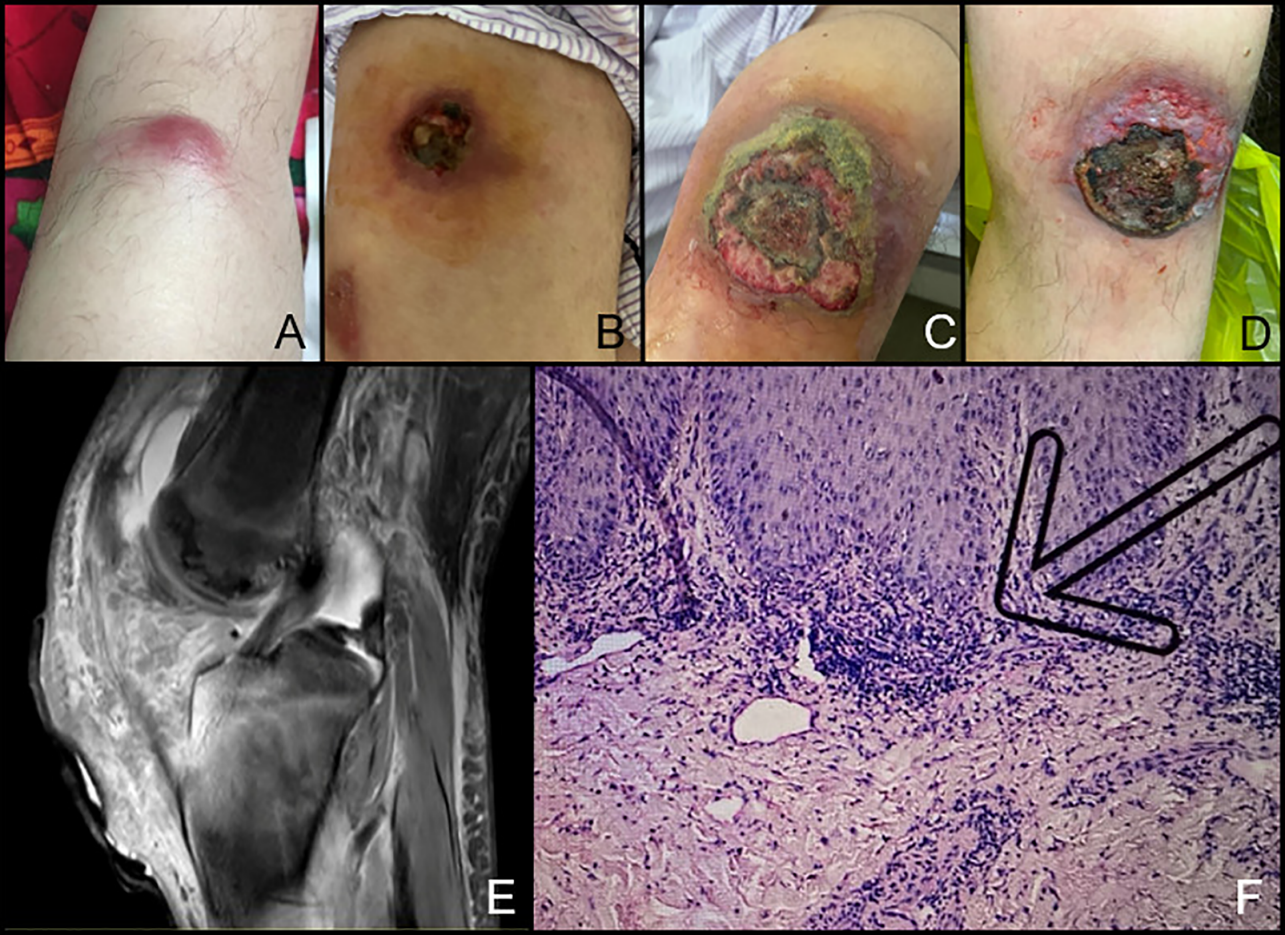
Skin lesion and arthritis of the Chinese patient with APLAID. **(A)** The lump under the left knee; **(B, C)**. The ulceration which was diagnosed as gangrenous pyoderma got worse. **(D)** Ulceration healing after dressing changing and adalimumab treatment. **(E)** Bone marrow erosion of the knee on MRI. **(F)** Skin biopsy of the ulceration. The arrow indicates perivascular lymphocyte infiltration.

He remained stable with only regular IVIG infusion for 6 months, when he noticed pitting edema in his left lower limb, followed by two lumps in his left leg and knee arthritis (**Figure 2A**). Two weeks later, the lumps got postulated with reddish-swelling, ulceration and effusion (**Figure 2B**). Antibiotics were ineffective, pustules and arthritis got worse (**Figure 2C**). Serum CRP level rose to 66.02 mg/L and IgE level was 779 IU/ml. MRI revealed increased bone marrow edema, joint fluid and soft tissue effusion around the knee (**Figure 2E**). Skin biopsy showed massive perivascular lymphocyte infiltration without signs of infection or lymphoma (**Figure 2F**). Gangrenous pyoderma was diagnosed by dermatologists. Synovial fluid revealed clear joint fluid with ample white blood cells but no pathogens. He was treated with prednisone of 60 mg per day, methotrexate 12.5 mg per week and thalidomide 25 mg per night. His arthralgia improved and serum CRP level and IgE level transiently decreased to 8.45 mg/dl and 395 U/L, respectively. But his skin ulceration remained unchanged. Soon, he began to have fever, and CRP (29.27 mg/dl) and IgE (853 U/L) level increased again. Owing to abnormal liver function, immunosuppressants were stopped and subcutaneous adalimumab 40 mg every 2 weeks was added. After the first dose, gangrenous pyoderma had greatly improved (**Figure 2D**) and CRP decreased to 6.19 mg/dl. Unfortunately, he got an EBV positive T cell lymphoma 2 months later, which was in accordance with immunodeficiency-associated lymphoproliferative disorders.

### Literature Review

Six English-language publications describing a total of 10 cases of APLAID were identified through the PubMed search (7–12). Due to its rarity there was no cohort study of APLAID yet. All 11 patients had heterozygous mutations in the *PLCG2* gene. The allele frequencies were as follows: M1141K 27%, S707Y 18%, L848P 18%, A708P 9%, L845_L848del 9%, H193Q 9%, and I169V 9%. The clinical phenotypes and genotypes of the 10 patients and our patient were listed in **Table 1**.

**Table 1:** Summarization of phenotypic and genotypic features of 11 patients with APLAID.

Characteristics	Zhou, et al.	Novice, et al	Khabbazi, et al.	Martín-Nald, et al.	Neves, et al.	Morán-Villaseñor, et al	Our study
Number of cases	2	3	1	2	1	1	1
Ethnicity	ND	ND	ND	ND	Portuguese	ND	Chinese
Country	America	ND	Iran	Spain	Portugal	Mexico	China
Gender	1F, 1M	3F	F	1F, 1M	F	M	M
Age at onset	Infancy^*^	2 infancy^*^, 1 ND	8 years old	Infancy^*^	7 days after birth	3 days after birth	5 years old
Age at diagnosis (years old)	ND	2, 6 (1ND)	11	16, 9	11	3	21
Family history	+	2+, 1-	−	−	−	−	−
Fever	−	−	+	−	−	−	+
Cutaneous granulomas	+	−	–	1+, 1-	–	+	–
Vesiculopustular rashes	+	2+, 1-	+	+	+	+	+
Cutis laxa	−	−	−	+	+	−	−
Arthralgia/arthritis	+	1+, 2-	+	−	+	−	+
Enterocolitis	+	1+, 2-	+	−	+	+	−
Eye inflammation	+	1+, 2-	−	+	+	+	−
Sinopulmonary infection	+	+	−	+	+	+	+
Interstitial pneumonitis	+	−	−	−	+	−	−
Sensorineural deafness	−	−	−	−	+	−	−
Headache	−	−	+	−	−	−	+
Humoral immunodeficiency							
Reduced IgM level	+	+	−	+	+	+	+
Reduced IgG level	−	1+, 2ND	−	+	+	+	+
Reduced IgA level	+	1+, 2ND	−	+	+	+	+
Decreased B cell count	+	2+, 1-	−	+	+	+	−
Decreased NK cell count	−	ND	–	–	–	+	−
Increased IgE level	ND	ND	−	−	ND	ND	+
Elevated ESR/CRP	ND	ND	+	+	ND	ND	+
*PLCG2* mutation	S707Y	M1141K	H193Q	L845_L848del A708P	L848P	L848P	I169V
Treatment							
Corticosteroids	+ (high dose)	+ (high dose), 2 ND	+ (high dose)	+	+ (high dose)	−	+ (high dose)
I mmunosuppressant	−	2−, 1ND	+ (MTX)	−	+ (HCQ)	+ (MTX)	+ (MTX, CsA)
Biologics	TNF inhibitor; IL-1 inhibitor	1+ (omalizumab;anakinra), 1-, 1ND	+ (etanercept)	1+ (etanercept, Anakinra), 1-	+ (anakinra, canakinumab,ruxolitinib)	+ (infliximab)	+ (adalimumab)
IVIG	−	2+, 1ND	−	+	−	+	+
Prognosis	ND	skin lesion improved, 2ND	without success	improved, a partial control of skin inflammation	partially improved	stable	stable

*The exact ages were not recorded.

ND, no data; Ig, immunoglobulin; IVIG, intravenous immunoglobulin; MTX, methotrexate; HCQ, hydroxychloroquine; CsA, cyclosporine.

The overall ratio of male to female was 4:7. Two patients were father and his daughter (8), and two were mother and her daughter (7). The rest patients had no family history of autoinflammatory diseases. Most patients had disease onsets during their infancies (5, 6, 8–10). The most frequently affected organs were skin (11/11, 100.0%), lung (10/11, 90.9%), joints (8/11, 72.7%), gastrointestinal tracts (8/11, 72.7%), and eyes (5/11, 45.5%). Skin manifestations included cutaneous granulomas, vesiculopustular rashes and cutis laxa (7, 10, 11). Gangrenous pyoderma has never been reported before. Pulmonary involvement featured as recurrent sinopulmonary infection and interstitial pneumonitis (7, 11). Gastrointestinal tract findings included ulcerative colitis and diarrhea (9, 11). 10 patients presented with immunodeficiency, including hypo-IgG (6/10, 60%), hypo-IgM (10/10, 100%), hypo-IgA (8/10, 80%), low B cells count (8/10, 80%), and low NK cells count (1/10, 10%) (5, 6, 8–10). Special clinical symptoms such as sensorineural deafness and central nervous system vasculitis had occasionally been reported (11, 12).

Of the eleven patients, eight (72.7%) have been treated with glucocorticoids, and inflammatory manifestations were partially ameliorated in five (45.5%) patients. However, the side effects limited the dosage and duration of glucocorticoids in two (18.2%) patients (7–12). TNFα inhibitors (including infliximab and etanercept) were given to four patients and only one patient showed partial response to infliximab (7, 9, 10). IL-1 inhibitors were tried in four patients and were only marginally effective in one patient (7, 8, 10, 11). Intravenous immunoglobulin infusion was administered in three patients for hypogammaglobulinemia (8, 10, 12). However, no patient had a complete recovery and all of them suffered from disease relapse.

## Discussion

APLAID is a rare autosomal dominant autoinflammatory disorder characterized by recurrent blistering skin lesions, and the wide phenotypic variability including eye inflammation, arthralgia, enterocolitis, interstitial pneumonitis and recurrent sinopulmonary infections, accompanied with immunodeficiency (7–12). Here, we described a young Chinese patient with recurrent fever, vesiculopustular skin lesions, arthritis, bronchitis, and immunodeficiency which manifested as decreased IgM, IgG, IgA, B, and NK cells counts. Whole exome sequencing variant filtering revealed a novel heterozygous I169V mutation in the *PLCG2* gene. According to gnomAD database, the Minor Allele Frequency (MAF) of this variant was 0.0013, and it is predicted to be damaging when using a variety of in silico pathogenicity prediction tools including MutationTaster, M-CAP, VEST3, Genocanyon, and FATHMM_MKL. Besides, sequence alignment of PLCγ2 among various vertebrates demonstrates that Ile169 is a highly conserved residue, indicating it may play a fundamental role in cell and any mutation may lead to dysfunction. Although we were not able to carry out functional studies, we suggest this *PLCG2* variant is associated with the phenotype of the patient, and diagnosis of APLAID could be confirmed due to the combination of characteristic phenotype and genotype results. To the best of our knowledge, this is the very first case of Chinese Han patient with APLAID, indicating that APLAID may occur in various ethnic groups worldwide. Intriguingly, we noticed a high serum IgE level in this patient, which had not been observed in APLAID patients before. No infections, autoimmune diseases, allergic diseases, and gene mutations associated with hyperimmunoglobulinemia E syndrome were found. His IgE level fluctuated with his systemic inflammation and his asymptomatic father also had increased IgE level, which suggests that increased IgE level might be related to his genomic abnormality. Interestingly, the patient’s father carried the same *PLCG2* mutation while presented no other symptoms except for the high IgE level. Similar to our case, it has been previously reported that parents of an APLAID patient were heterozygous without any manifestation of the disease despite the dominant inheritance of this syndrome (9). We inferred that incomplete penetrance of the *PLCG2* mutation might explain why the father with the same mutation didn’t exhibit any clinical signs of the disorder (13). On the other side, in a recent study, IP_3_-mediated Ca^2+^ release from the endoplasmic reticulum was testified to enhance NLRP3 inflammasome activation in peripheral blood mononuclear cells (PBMCs) from APLAID patients, suggesting that NLRP3 inflammasome activation may play a role in the pathogenesis of APLAID (14). In addition, ex vivo experiments showed increased amount of Ca^2+^ into cytosol upon crosslinking stimulation with IgE in PBMCs from APLAID patients, which may then trigger NLRP3 inflammasome activation (7). These data suggested that the IgE might contribute to the inflammation in APLAID disease. Further studies are needed to explore the mechanism of hyper-IgE and its relation to APLAID. Nevertheless, our report expanded the phenotype of APLAID and demonstrated the disease heterogeneity among individuals.

It is essential to distinguish APLAID from another autosomal dominant inherited disease, PLCγ2-associated antibody deficiency and immune dysregulation (PLAID), owing to deletions of the *PLCG2* gene (15). In contrast to APLAID, which is caused by substitutions of the *PLCG2* gene and gain-of-mutation of the protein (7–12), PLAID is caused by exon deletions and loss-of-function of the protein. The two disorders have overlapping clinical manifestations like inflammation and immune deficiency. However, PLAID is characterized by cold-induced urticaria, allergic diseases, positive ANAs (up to 2/3 of the patients), and hyper-IgE (16, 17).

To date, there is no standard management strategy for APLAID. High-dose corticosteroids were partially effective in some patients but the dosage and duration of treatment have been limited by side effects. IVIG was given for hypogammaglobulinemia. Similar to other autoinflammatory diseases, biologics served as a potential therapy, yet the effectiveness was not obvious. Early hematopoietic stem-cell transplantation was considered to improve the condition (11, 12), but no patient received such therapy till now. In this patient, medium dose prednisone and MTX, along with regular IVIG infusion were effective in controlling his papulosis rashes and arthritis, but gangrenous pyoderma showed minimal response. In a pooled analysis of gangrenous pyoderma, medium to large dose of corticosteroids and CsA both had received a 47% of healing rate and a 28% to 30% of recurrence rate. Since a previous study showed that PBMCs from patients with APLAID were found to secrete IL-1β in response to LPS priming (13), IL-1 inhibitors may be considered to treat APLAID. Nonetheless, as mentioned above, only one patient was once reported to have marginal response to IL-1β inhibitors (7, 8, 10, 11). Meanwhile, recent data confirmed the definite effectiveness of TNFα inhibitors in patients with gangrenous pyoderma, with the responding rate reached up to 82% to 100%, and the healing rate reached 40% to 50% (18, 19). Among these TNFα inhibitors, adalimumab showed the best treatment effects (18). Considering the persistent liver damage in our patient, we tried adalimumab in this patient and found a good response. However, the patient had EBV infection during the treatment and soon got T cell lymphoma which was associated with the immunodeficiency.

This case report is limited by the fact that we did not perform functional studies for the defined variant. However, using a variety of silico analysis algorithm, this variant was predicted to be damaging. Thus, we suggest that this variant is associated with the phenotype of the patient, and we hope further studies will enlighten the underlying mechanisms, particularly its relationship with increased IgE level. Second, treatment with adalimumab was only empirical. Long-term follow-up was needed to evaluate the effect of adalimumab.

In conclusion, APLAID is a newly-defined autoinflammatory disease. Due to its rarity and heterogeneous clinical manifestations, it is difficult to be diagnosed and treated. Our study reported the first case of APLAID with gangrenous pyoderma and concomitant high IgE carrying a novel *PLCG2* mutation, which may expand the clinical phenotype and genotype of APLAID. TNFα inhibitors could be considered in APLAID patient with gangrenous pyoderma.

## Data Availability Statement

The original contributions presented in the study are included in the article, further inquiries can be directed to the corresponding author.

## Ethics Statement

Written informed consent was obtained from the individual for the publication of any potentially identifiable images or data included in this article.

## Author Contributions

All authors listed have made a substantial, direct, and intellectual contribution to the work and approved it for publication.

## Funding

This work was supported by the Natural Science Foundation of Beijing (Grant No. 7192170), the Chinese Academy of Medical Sciences Innovation Fund for Medical Sciences (CIFMS) (Grant No. 2017-I2M-3-001), Fundamental Research Funds for the Central Universities (Grant No. APL20100310010301019), and the National Key Research and Development Program of China (Grant No. 2016YFC0901500; 2016YFC0901501).

## Conflict of Interest

The authors declare that the research was conducted in the absence of any commercial or financial relationships that could be construed as a potential conflict of interest.

## References

[1] Krainer J Siebenhandl S Weinhäusel A. Systemic Autoinflammatory Diseases. J Autoimmun (2020) 109:102421. 10.1016/j.jaut.2020.102421 32019685 PMC7610735

[2] McDermott MF Aksentijevich I Galon J McDermott EM Ogunkolade BW Centola M. Germline Mutations in the Extracellular Domains of the 55 Kda TNF Receptor, TNFR1, Define a Family of Dominantly Inherited Autoinflammatory Syndromes. Cell (1999) 97:133–44. 10.1016/s0092-8674(00)80721-7 10199409

[3] Manthiram K Zhou Q Aksentijevich I Kastner DL. The Monogenic Autoinflammatory Diseases Define New Pathways in Human Innate Immunity and Inflammation. Nat Immunol (2017) 18:832–42. 10.1038/ni.3777 28722725

[4] Hurley JH Grobler JA. Protein Kinase C and Phospholipase C: Bilayer Interactions and Regulation. Curr Opin Struct Biol (1997) 7:557–65. 10.1016/s0959-440x(97)80122-4 9266179

[5] Hiller G Sundler R. Regulation of Phospholipase C-Gamma 2 *Via* Phosphatidylinositol 3-Kinase in Macrophages. Cell Signal (2002) 14:169–73. 10.1016/s0898-6568(01)00252-2 11781142

[6] Everett KL Bunney TD Yoon Y Rodrigues-Lima F Harris R Driscoll PC. Characterization of Phospholipase C Gamma Enzymes With Gain-of-Function Mutations. J Biol Chem (2009) 284:23083–93. 10.1074/jbc.M109.019265 PMC2755714 19531496

[7] Zhou Q Lee GS Brady J Datta S Katan M Sheikh A. A Hypermorphic Missense Mutation in PLCG2, Encoding Phospholipase Cc2, Causes a Dominantly Inherited Autoinflammatory Disease With Immunodeficiency. Am J Hum Genet (2012) 91:713–20. 10.1016/j.ajhg.2012.08.006 PMC3484656 23000145

[8] Novice T Kariminia A Del Bel KL Lu H Sharma M Lim CJ. A Germline Mutation in the C2 Domain of Plcγ2 Associated With Gain-of-Function Expands the Phenotype for PLCG2-Related Diseases. J Clin Immunol (2020) 40:267–76. 10.1007/s10875-019-00731-3 PMC7086538 31853824

[9] Khabbazi A Rahbar Kafshboran H Nasiri Aghdam M Nouri Nojadeh J Daghagh H Daneshmandpour Y. A New Report of Autoinflammation and PLCG2-Associated Antibody Deficiency and Immune Dysregulation (APLAID) With a Homozygous Pattern From Iran. Immunol Lett (2020) 221:27–32. 10.1016/j.imlet.2020.01.008 32014489

[10] Martín-Nalda A Fortuny C Rey L Bunney TD Alsina L Esteve-Solé A. Severe Autoinflammatory Manifestations and Antibody Deficiency Due to Novel Hypermorphic PLCG2 Mutations. J Clin Immunol (2020) 40:987–1000. 10.1007/s10875-020-00794-7 32671674 PMC7505877

[11] Neves JF Doffinger R Barcena-Morales G Martins C Papapietro O Plagnol V. Novel PLCG2 Mutation in a Patient With APLAID and Cutis Laxa. Front Immunol (2018) 9:2863. 10.3389/fimmu.2018.02863 30619256 PMC6302768

[12] Morán-Villaseñor E Saez-de-Ocariz M Torrelo A Arostegui JI Yamazaki-Nakashimada MA Alcántara-Ortigoza MA. Expanding the Clinical Features of Autoinflammation and Phospholipase Cγ2-Associated Antibody Deficiency and Immune Dysregulation by Description of a Novel Patient. J Eur Acad Dermatol Venereol (2019) 33:2334–39. 10.1111/jdv.15918 31465591

[13] Taeubner J Wieczorek D Yasin L Brozou T Borkhardt A Kuhlen M. Penetrance and Expressivity in Inherited Cancer Predisposing Syndromes. Trends Cancer (2018) 4:718–28. 10.1016/j.trecan.2018.09.002 30352675

[14] Chae JJ Park YH Park C Hwang IY Hoffmann P Kehrl JH. Connecting Two Pathways Through Ca^2+^ Signaling: NLRP3 Inflammasome Activation Induced by a Hypermorphic PLCG2 Mutation. Arthritis Rheumatol (2015) 67:563–7. 10.1002/art.38961 PMC4369162 25418813

[15] Ombrello MJ Remmers EF Sun G Freeman AF Datta S Torabi-Parizi P. Cold Urticaria, Immunodeficiency, and Autoimmunity Related to PLCG2 Deletions. N Engl J Med (2012) 366:330–8. 10.1056/NEJMoa1102140 PMC3298368 22236196

[16] Shea J Huynh T Milner J Chamlin S. PLAID Syndrome: Characteristic Presentation and a Novel Therapeutic Option. Pediatr Dermatol (2020) 37:147–9. 10.1111/pde.13972 31633221

[17] Milner JD. PLAID: A Syndrome of Complex Patterns of Disease and Unique Phenotypes. J Clin Immunol (2015) 35:527–30. 10.1007/s10875-015-0177-x PMC4575258 26206677

[18] Quist SR Kraas L. Treatment Options for Pyoderma Gangrenosum. J Dtsch Dermatol Ges (2017) 15:34–40. 10.1111/ddg.13173 28140549

[19] Abdallah HB Fogh K Bech R. Pyoderma Gangrenosum and Tumor Necrosis Factor Alpha Inhibitors: A Semi-Systemic Review. Int Wound J (2019) 16:511–21. 10.1111/iwj.13067 PMC7949186 30604927

